# Intraoperative hypovolemia as a possible precipitating factor for pituitary apoplexy: a case report

**DOI:** 10.1186/s13256-022-03738-4

**Published:** 2023-02-10

**Authors:** Kristine M. Abo, Joseph Kane, Rachel C. Druckenbrod, Michael A. Mooney, Jacob Rachlin

**Affiliations:** 1 Boston University School of Medicine, Boston, MA USA; 2 Department of Optometry, VA Boston Healthcare System, Jamaica Plain, Boston, MA USA; 3 Neurosurgery Division, Department of Surgery, VA Boston Healthcare System, Jamaica Plain, Boston, MA USA; 4 Department of Neurosurgery, Brigham and Women’s Hospital, Harvard Medical School, Boston, MA USA

**Keywords:** Pituitary apoplexy, Pituitary adenoma, Ophthalmoplegia, Case report

## Abstract

**Background:**

Pituitary apoplexy is acute infarction with or without hemorrhage of the pituitary gland. It is a rare but potentially life-threatening emergency that most commonly occurs in the setting of pituitary adenoma. The mechanisms underlying pituitary apoplexy are not well understood, but are proposed to include factors of both hemodynamic supply and adenoma demand. In the case of patients with known pituitary macroadenomas undergoing major surgery for other indications, there is a theoretically increased risk of apoplexy in the setting of “surgical stress.” However, risk stratification of patients with nonfunctioning pituitary adenomas prior to major surgery is challenging because the precipitating factors for pituitary apoplexy are not completely understood. Here we present a case in which intraoperative hypovolemia is a possible mechanistic precipitating factor for pituitary apoplexy.

**Case presentation:**

A 76-year-old patient with a known hypofunctioning pituitary macroadenoma underwent nephrectomy for renal cell carcinoma, during which there was significant intraoperative blood loss. He became symptomatic with ophthalmoplegia on the second postoperative day, and was diagnosed with pituitary apoplexy. He was managed conservatively with cortisol replacement therapy, and underwent therapeutic anticoagulation 2 months after pituitary apoplexy for deep vein thrombosis. His ophthalmoplegia slowly resolved over months of follow-up. Pituitary apoplexy did not recur with therapeutic anticoagulation.

**Conclusions:**

When considering the risk of surgery in patients with a known pituitary macroadenoma, an operation with possible high-volume intraoperative blood loss may have increased risk of pituitary apoplexy because intraoperative hypovolemia may precipitate ischemia, infarction, and subsequent hemorrhage. This may be particularly relevant in the cases of elective surgery. Additionally, we found that we were able to therapeutically anticoagulate a patient 2 months after pituitary apoplexy for the management of deep vein thrombosis without recurrence of pituitary apoplexy.

## Background

Pituitary apoplexy is acute infarction with or without hemorrhage of the pituitary gland. It is a rare and potentially life-threatening emergency that most commonly occurs in the setting of pituitary adenoma. Postoperative pituitary apoplexy has been described after lobectomy [14], lumbar fusion [1, 8], coronary artery bypass grafting [7], thyroidectomy [9], and arthroplasty [10]. Perioperative factors contributing to apoplexy are not known, with theories ranging from characteristics of the adenoma such as abnormal vasculature, large size, and growth velocity, to changes in hemodynamics in the setting of surgery resulting in adenoma ischemia or predisposing to hemorrhage. Here, we report a case of pituitary apoplexy in a patient with a nonfunctioning pituitary macroadenoma following nephrectomy with high-volume blood loss. Significant intraoperative blood loss may have precipitated pituitary apoplexy in this patient via pituitary hypoperfusion. Subsequent conservative management was hemodynamically complicated by deep vein thrombosis, and the patient was anticoagulated without recurrence of pituitary hemorrhage.

## Case report

### Initial presentation

**Fig. 1:**
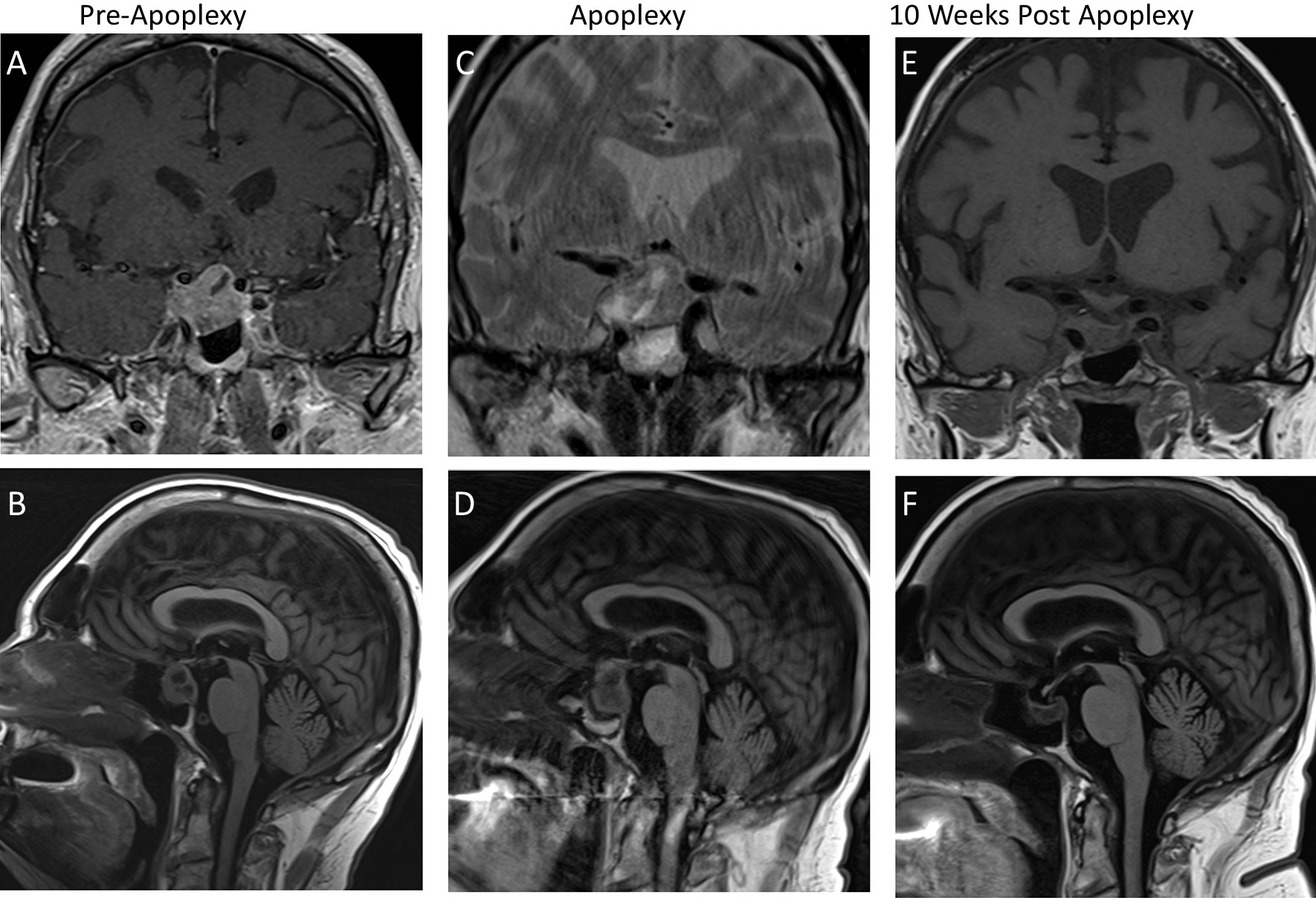
Preoperative MRI shows pituitary macroadenoma (2.7 cm) with suprasellar extension and compression of optic chiasm, parasellar extension into right cavernous sinus, and extension into right sphenoid sinus on coronal T1 with contrast (**a**) and sagittal T2 FLAIR (**b**). Postoperative day 7 MRI demonstrates hemorrhage on coronal T2 (**c**) and sagittal T2 FLAIR (**d**). Postoperative week 10 MRI demonstrating resolution of hemorrhagic changes within pituitary gland, decrease in size of adenoma now limited to sella, and no mass effect on optic chiasm on coronal T1 without contrast (**e**) and sagittal T2 FLAIR (**f**)

A 76-year-old man with a history of renal cell carcinoma with prior left nephrectomy and chronic kidney disease presented with low libido and erectile dysfunction ongoing for several years. Laboratory evaluation revealed low testosterone, luteinizing hormone, and follicle stimulating hormone levels, consistent with hypogonadotropic hypogonadism (Table 1). This prompted brain magnetic resonance imaging (MRI), which demonstrated a large sellar mass (up to 2.7 cm in diameter) with suprasellar extension, optic chiasmal contact, and extension into the right sphenoid and cavernous sinuses (Fig. 1A, B). The patient was diagnosed with a nonfunctioning pituitary macroademona with partial hypopituitarism (Table 1). At this time, despite radiological evidence of optic chiasm compression and cavernous sinus extension, the patient had no clinical evidence of a sellar mass effect, including intact visual fields, normal funduscopic appearance of the optic nerves, and absence of ophthalmoplegia bilaterally. He had no symptoms of hypopituitarism other than sexual dysfunction.

**Table 1:** Laboratory evaluation

Analyte	Preoperative level	Preoperative interpretation	Postoperative level	Postoperative interpretation
Total testosterone (AM)	149.81 ng/dl	Low		
Free testosterone	15.7 ng/dl	Low		
Luteinizing hormone	0.99 mIU/ml	Low	0.32 mIU/ml	Low
Follicle stimulating hormone	4.01 mIU/ml	Low normal	2.3 mIU/ml	Low
Prolactin	26.8 ng/ml	High	10 ng/ml	Normal
Thyroid stimulating hormone	0.39 mlU/ml	Low	0.02 mIU/ml	Low
Free T4	0.9 ng/dl	Low normal	0.81 ng/dl	Low
Growth hormone	< 0.1 ng/dl	Low		
Insulin-like growth factor 1	60 ng/ml	Normal	81 ng/ml	Normal
Cortisol (AM)	5.84 μg/dl	Low	5.19 μg/dl	Low
Adrenocorticotropic hormone	26 pg/ml	Normal

Concurrently, abdominal MRI identified a right kidney mass (1.4 × 3.4 × 5.6 cm) concerning for recurrent malignancy. Given high suspicion for malignancy in the solitary kidney in the setting of chronic kidney disease, a partial right nephrectomy was planned. Transphenoidal resection of the pituitary adenoma was deferred at this time.

### Nephrectomy and postoperative course

The patient underwent partial right nephrectomy with pathological confirmation of clear cell renal carcinoma. Given the patient’s low preoperative morning cortisol level (Table 1), the patient was treated with 2 days of intravenous corticosteroid supplementation beginning the day of surgery. The intraoperative course was complicated by bleeding at the surgical site with significant blood loss (estimated 3 L) requiring 3 units packed red blood cells and 4 L fluid replacement intraoperatively, as well as brief vasopressors postoperatively. The period of hypovolemia was likely significant, as the patient had postoperative anuria ultimately requiring ureteric stenting on postoperative day 5.

On postoperative day 2, the patient reported new-onset moderate headache and near-complete right-sided ptosis was observed. When the right eyelid was lifted, he experienced binocular diplopia. Examination showed severe right-eye ophthalmoplegia (80–90% limitations in adduction, elevation, and depression, and complete limitation of abduction), mild right pupillary mydriasis with normal reactivity, and no relative afferent pupillary defect. Visual acuity was 20/50 in the right eye (reduced from 20/20), though color vision was intact, visual fields were full by confrontation field testing, and the structural eye examination was normal. He was diagnosed with right-sided pupil-involving partial third nerve palsy, complete sixth nerve palsy, and possible early compressive optic neuropathy.

Postoperative laboratory evaluation revealed panhypopituitarism (Table 1) and intravenous corticosteroid supplementation was resumed. Repeat brain MRI on postoperative day 7 was performed without contrast (in the setting of acute-on-chronic kidney injury with solitary partial kidney) and demonstrated increased heterogeneity of the sellar and suprasellar mass with areas of predominantly right-sided hemorrhage, consistent with expected radiographic changes observed in the days following pituitary apoplexy [4]. Extension to the optic chiasm, right sphenoid sinus, and right cavernous sinus (Fig. 1C, D) was unchanged. The patient was transitioned to oral corticosteroid replacement therapy with close monitoring of visual parameters.

### Follow-up care and monitoring

By postoperative day 10 (after 48 hours of oral corticosteroid therapy), visual acuity improved to baseline and ophthalmoplegia improved with reduced extent of cranial nerve 3 palsy, though cranial nerve 6 palsy remained complete. On postoperative day 11, the patient was discharged with continued corticosteroid replacement therapy. At 6 weeks post nephrectomy, the patient’s visual acuity remained normal and visual fields remained full by formal automated testing (Humphrey 24-2). His third nerve palsy fully resolved by postoperative week 6 while the sixth nerve palsy did not fully resolve until postoperative month 5. Throughout his course he showed no evidence of optic neuropathy by funduscopic examination nor by ophthalmic imaging with optical coherence tomography.

### Anticoagulation without recurrence of apoplexy

On a subsequent admission 2 months after nephrectomy, the patient was found to have left lower extremity deep vein thromboses (DVT) without evidence of pulmonary embolism on computed tomography pulmonary angiogram. Repeat brain MRI showed resolution of pituitary adenoma hemorrhage with partial involution (Fig. 1E, F), and the patient was administered therapeutic anticoagulation. The patient did not develop further clinically evident DVT, and his pituitary apoplexy did not recur.

## Discussion

Although pituitary apoplexy most commonly occurs in the setting of a known pituitary adenoma, it remains a rare condition with a risk of 0.2–0.6 events per 100 person-years among patients with known nonfunctioning pituitary adenomas [6]. Surgical intervention for nonfunctioning adenomas is generally reserved for patients who become symptomatic, most commonly through mass effect rather than via hypoandrogenism, and transsphenoidal resection is first-line treatment in these cases. However, the selection of patients for conservative management remains an area of investigation as the precipitating factors for pituitary apoplexy are not completely understood [3]. In particular, there are no guidelines that enable risk stratification of patients with nonfunctioning pituitary adenomas for postoperative pituitary apoplexy.

It is thought that pituitary apoplexy can result from multiple pathophysiological mechanisms that ultimately cause ischemia. These include factors that contribute to outgrowth of blood supply (the small arteries from the medial wall of the cavernous carotid), such as the presence of a pituitary tumor or pituitary hyperplastic states such as pregnancy or exogenous estrogen intake. Factors that contribute to reduced blood flow may also contribute, such as acute hypotension or thromboembolic events in perturbed hemostasis. Additionally, pituitary adenomas themselves may have abnormal vasculature that renders them intrinsically susceptible to infarction. In the case presented here, it is plausible that a combination of increased hemodynamic demand by the macroadenoma, adenoma-intrinsic susceptibility, and coagulopathy of chronic kidney disease played combined predisposing roles, while intraoperative blood loss resulted in decreased vascular flux and ultimately precipitated pituitary ischemia followed by infarction, necrosis, and subsequent hemorrhage. This case suggests that the risk of pituitary apoplexy may be higher in patients with known adenomas undergoing a procedure with a high risk of intraoperative blood loss.

Pituitary apoplexy most commonly presents with a sudden-onset, severe headache; visual defects; and ophthalmoplegia [6]. Ophthalmic signs are not always present initially [10] and their absence should not preclude evaluation for hypopituitarism. Although deficiency in any pituitary hormone can occur, adrenocorticotropin deficiency is critical to identify because it can lead to cardiovascular collapse, which is preventable through administration of corticosteroids. Our patient received perioperative intravenous corticosteroid supplementation and 3 weeks of oral corticosteroid supplementation. Our patient’s presentation with ophthalmoplegia on the second postoperative day fits the time course that would occur with necrosis and secondary hemorrhage.

Our case was further complicated by the question of whether prior pituitary apoplexy without resection is a contraindication for anticoagulation in the management of deep vein thrombosis. Anticoagulation itself can precipitate pituitary apoplexy [5, 11–13], but it is not known whether anticoagulation after pituitary apoplexy increases the risk of recurrent hemorrhage. Here, given radiographic evidence of pituitary adenoma hemorrhage resorption and involution, and the high risk of further deep vein thrombosis development, the patient was given therapeutic anticoagulation to prevent further thrombosis. The patient showed no signs of recurrent pituitary apoplexy, and continued to recover extraocular movement at frequent follow-ups with optometry. The patient will continue to be monitored with serial eye exams and neuroimaging.

## Conclusions

When considering the risk of surgery in patients with a known pituitary macroadenoma, an operation with possible high-volume intraoperative blood loss may have increased risk of pituitary apoplexy because intraoperative hypovolemia may precipitate ischemia, infarction, and subsequent hemorrhage. This may be particularly relevant in the cases of elective surgery. Additionally, we found that we were able to therapeutically anticoagulate a patient 2 months after pituitary apoplexy for the management of DVT without recurrence of pituitary apoplexy.

## Data Availability

Data sharing is not applicable to this article as no datasets were generated or analyzed during the current study.

## Abbreviations

DVT: Deep vein thrombosis
MRI: Magnetic resonance imaging
